# Direct analysis of HLA/HER-2 peptide epitopes

**DOI:** 10.1186/2051-1426-1-S1-P62

**Published:** 2013-11-07

**Authors:** Andrea Patterson, Wilfried Bardet, Ken Jackson, Jon Weidanz, William Hildebrand

**Affiliations:** 1 University of Oklahoma Health Sciences Center, Oklahoma City, OK, USA; 2 Texas Tech University Health Sciences Center, Lubbock, TX, USA

## Background

T cells recognize cancer cells via human leukocyte antigen (HLA)/peptide complexes, and class I HLA restricted tumor infiltrating T cells correspond to favorable prognoses in several cancers, including ovarian cancer. Cancer-specific HLA/peptide complexes therefore represent tumor cell surface biomarkers, and the accurate identification of HLA/peptide targets is a prerequisite for the development of T cell therapies. Potential class I HLA presented peptide fragments of human epidermal growth factor receptor 2 (HER-2) have been identified using both predictive algorithms and immunologic testing of HER-2 candidate peptides, including the controversial HER-2 peptide known as E75. Here, HLA were harvested directly from ovarian cancer cells and their peptide cargo assessed in order to determine which HER-2 peptides, including E75, represent true HLA tumor ligands.

## Methods and results

A secreted form of HLA-A*02:01 was stably transfected into the immortalized normal ovarian epithelial cell line FHIOSE-118 as well as into the SKOV-3 ovarian cancer cell line which overexpresses HER-2. HLA class I was harvested from both cell lines, the bound peptides compared by mass spectroscopy, and two HER-2-derived peptides were found unique to SKOV-3. One of these two peptides has been reported to bind HLA-A*02:01 and to be immunogenic, while the second HER-2 peptide represents a previously uncharacterized ligand. This novel HER-2 ligand was tested by competitive binding assay and found to have a high HLA-A*02:01 binding affinity. Interestingly, the widely accepted HER-2 peptide E75 was not present in the SKOV-3 peptide pool. To determine if another peptide ligand might be immunologically cross-reactive with E75, we tested an antibody that recognizes HLA-A*02:01/E75 for cross reactivity with other HLA-A*02:01/peptide complexes in the SKOV-3 line. Several HLA presented peptides represent potential E75 analogs.

## Conclusion

Class I HLA gathered from tumor lines provide for the direct identification of cancer-specific T cell biomarkers. Here, HLA class I gathered from ovarian cancer lines confirms that both expected and novel HER-2 peptides are available for T cell recognition, although the HER-2 peptides available for T cell targeting are not necessarily those predicted by indirect methods. In regards to the HLA-A*02:01/E75 ligand complex, our data coincide with published data showing that neither the standard nor the immunoproteasome are able to produce the E75 peptide. Gathering HLA from cancerous cells may provide more accurate knowledge of cancer epitopes, data paramount to their application either as prognostic biomarkers or in therapeutics such as peptide vaccination, antibody therapy, or adoptive cell transfer.

